# Baseline 25-hydroxyvitamin D and progression-free survival in de novo metastatic hormone-sensitive prostate cancer: prognostic model development and external validation

**DOI:** 10.1093/oncolo/oyag396

**Published:** 2026-10-06

**Authors:** Galip Can Uyar, Kadriye Başkurt, Orhun Akdoğan, Enes Yeşilbaş, Mustafa Ersoy, Tuba Uğur Tuzcu, Kadriye Bir Yücel, Mustafa Altınbaş, Ömür Berna Çakmak Öksüzoğlu, Gökşen İnanç İmamoğlu, Osman Sütcüoğlu

**Affiliations:** Department of Medical Oncology, Etlik City Hospital, Ankara, 06170, Turkey; Department of Medical Oncology, Etlik City Hospital, Ankara, 06170, Turkey; Department of Medical Oncology, Faculty of Medicine, Gazi University, Ankara, 06500, Turkey; Department of Medical Oncology, Etlik City Hospital, Ankara, 06170, Turkey; Department of Medical Oncology, Kütahya City Hospital, Kütahya, 43100, Turkey; Department of Medical Oncology, Faculty of Medicine, Gazi University, Ankara, 06500, Turkey; Department of Medical Oncology, Etlik City Hospital, Ankara, 06170, Turkey; Department of Medical Oncology, Etlik City Hospital, Ankara, 06170, Turkey; Department of Medical Oncology, Etlik City Hospital, Ankara, 06170, Turkey; Department of Medical Oncology, Etlik City Hospital, Ankara, 06170, Turkey; Department of Medical Oncology, Faculty of Medicine, Gazi University, Ankara, 06500, Turkey

**Keywords:** androgen receptor pathway inhibitors, metastatic hormone-sensitive prostate cancer, nomogram, prognostic biomarker, 25-hydroxyvitamin D

## Abstract

**Background:**

Prognostic biomarkers for risk stratification remain limited in *de novo* metastatic hormone-sensitive prostate cancer (mHSPC) treated with androgen receptor pathway inhibitors (ARPIs). We evaluated baseline serum 25-hydroxyvitamin D [25(OH)D] and developed and externally validated a progression-free survival (PFS) model.

**Methods:**

This multicenter retrospective study included 273 patients initiating ARPI-based therapy between January 2021 and April 2026. Etlik City Hospital formed the training cohort (*n* = 192; 85 PFS events), and Gazi University and Kütahya City Hospital formed the external validation cohort (*n* = 81; 27 PFS events). LASSO-penalized Cox regression retained age, ISUP grade group, CHAARTED disease volume, log-transformed PSA, and hemoglobin. Model B added continuous 25(OH)D. Internal validation used 1000 bootstrap resamples. Performance was assessed by the Harrell C-index, time-dependent AUC, and calibration at 1 and 2 years. PFS was the primary endpoint; overall survival analyses were exploratory. The study was not prospectively registered.

**Results:**

Higher baseline 25(OH)D was associated with longer PFS in Model B (HR per 10-ng/mL increase, 0.64; 95% CI, 0.49-0.83; *P* < .001). Adding 25(OH)D improved model fit (likelihood-ratio χ² = 12.71; *P* < .001) and increased apparent C-index from 0.712 to 0.740. In external validation, C-indices were 0.71 (95% CI, 0.64-0.78) and 0.76 (95% CI, 0.69-0.83), respectively (Δ = 0.05; 95% CI, 0.01-0.10).

**Conclusions:**

Baseline 25(OH)D added modest prognostic information beyond clinical factors, with supportive external validation. These findings do not establish treatment-predictive utility or benefit from vitamin D supplementation. Prospective validation is needed before clinical implementation.

## Graphical Abstract here

**Graphical Abstract:**
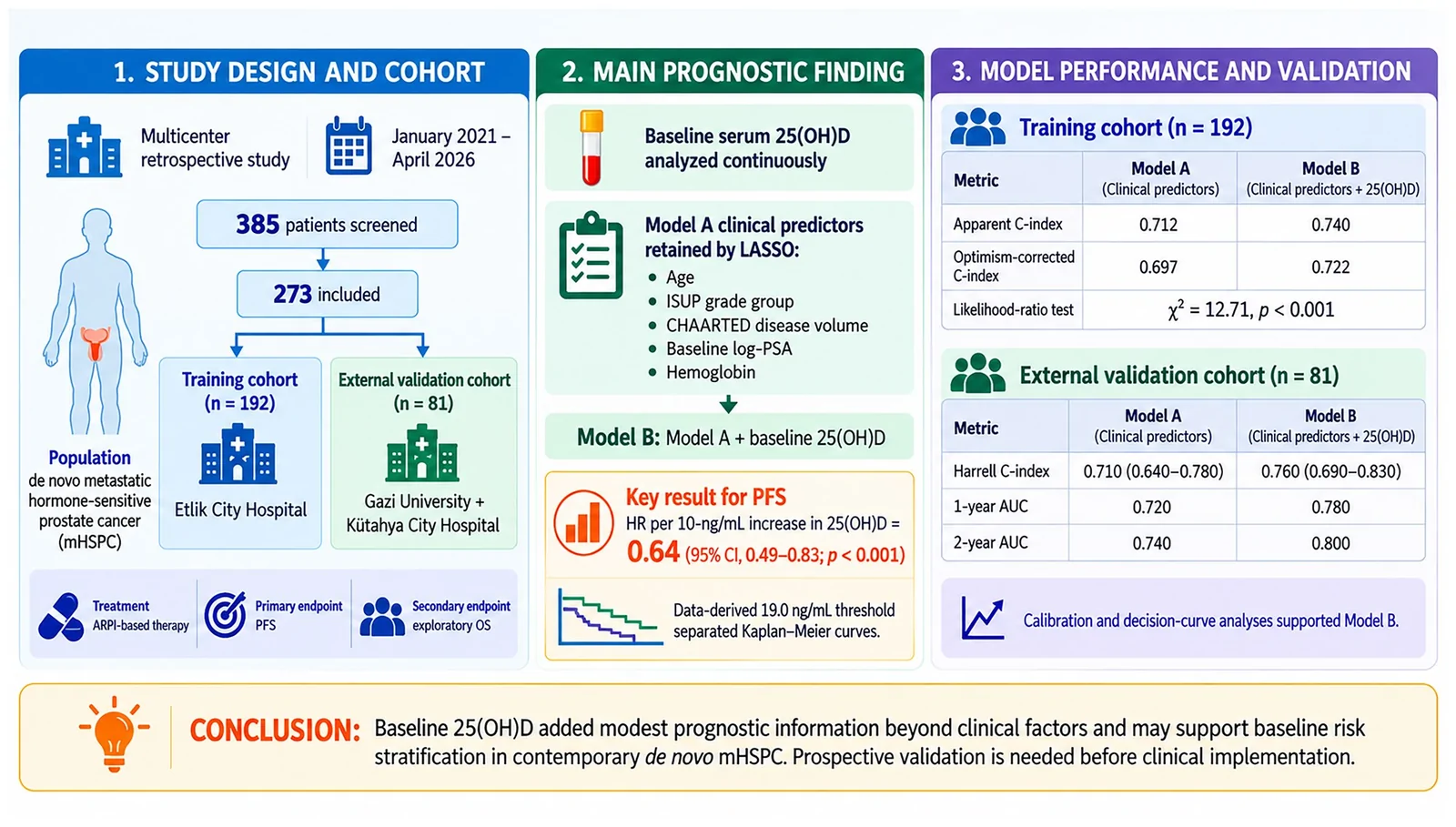

Implications for practice Baseline serum 25-hydroxyvitamin D may provide additional prognostic information in patients with *de novo* metastatic hormone-sensitive prostate cancer receiving contemporary androgen receptor pathway inhibitor therapy. In this multicenter study, higher baseline 25(OH)D was associated with longer progression-free survival, and its addition to a clinical prognostic model provided modest incremental information that was supported in an independent validation cohort. These findings support further evaluation of 25(OH)D as an accessible prognostic marker, but they do not demonstrate that vitamin D supplementation improves cancer outcomes or that vitamin D status should guide treatment selection.

## Introduction

Prostate cancer (PC) remains a major cause of cancer-related morbidity and mortality worldwide, and metastatic disease accounts for the greatest burden of lethal outcomes.^1^ The treatment landscape of metastatic hormone-sensitive prostate cancer (mHSPC) has changed substantially with the addition of androgen receptor pathway inhibitors (ARPIs), including abiraterone, enzalutamide, and apalutamide, to androgen deprivation therapy (ADT).^2–4^ Although contemporary treatment intensification has improved progression-free survival (PFS) and overall survival (OS), clinical outcomes remain heterogeneous, with some patients experiencing early progression despite ARPI-based therapy.^2–5^ Accurate baseline identification of patients at increased risk of progression may therefore complement established clinicopathological assessment and improve prognostic stratification at treatment initiation.^6^^,^^7^

Circulating 25-hydroxyvitamin D [25(OH)D], the principal biomarker of vitamin D status, has emerged as a potential prognostic factor in PC. Experimental studies have shown that vitamin D signaling may influence cellular proliferation, apoptosis, inflammation, and androgen receptor-related pathways,^8^^,^^9^ while circulating 25(OH)D also reflects aspects of nutritional and systemic inflammatory status.^10^ Clinical observational studies and meta-analyses have reported associations between lower circulating 25(OH)D levels and adverse pathological features or poorer survival outcomes in PC.^10–13^ However, interventional studies of vitamin D supplementation have produced inconsistent findings,^14^^,^^15^ and the recent randomized ProsD trial did not demonstrate reduced PC progression with vitamin D supplementation.^16^ Moreover, much of the available clinical evidence derives from localized PC, metastatic castration-resistant disease, or populations treated before widespread contemporary treatment intensification, and available studies often lack independent external validation.^11–13^^,^^17^ Thus, whether baseline 25(OH)D provides prognostic information beyond established clinical factors in patients with *de novo* mHSPC receiving contemporary ARPI-based therapy remains uncertain.

In the current study, we sought to determine whether baseline serum 25(OH)D provides incremental prognostic information beyond established clinical factors for PFS in patients with *de novo* mHSPC initiating ARPI-based therapy. We developed a clinical prognostic model and evaluated the incremental contribution of continuous baseline 25(OH)D, followed by independent external validation and complementary sensitivity analyses. We hypothesized that baseline 25(OH)D would provide additional prognostic information beyond routinely available clinicopathological factors. By addressing this question, our study aims to clarify the potential role of baseline 25(OH)D as a complementary marker for prognostic risk stratification in contemporary *de novo* mHSPC.

## Methods

### Study design and patient population

We conducted a multicenter retrospective real-world cohort study including patients with mHSPC who initiated ARPI-based therapy between January 2021 and April 2026 across 3 tertiary referral centers in Turkey. Follow-up continued through May 2026. The training cohort was derived from the Medical Oncology Department of Etlik City Hospital, Ankara, whereas the external validation cohort comprised patients treated at the Gazi University Medical Oncology Department, Ankara, and Kütahya City Hospital Medical Oncology Clinic. Etlik City Hospital, which contributed the largest single-center cohort, was used for model development, whereas patients from the 2 independent institutions constituted the external validation cohort.

Eligible patients had histologically confirmed prostate adenocarcinoma, radiologically confirmed metastatic disease at initial diagnosis, and initiated abiraterone, enzalutamide, or apalutamide in combination with continuous ADT. Patients with metastatic recurrence after prior treatment for localized disease, unavailable baseline serum 25(OH)D, insufficient baseline clinical or laboratory data, or insufficient follow-up information were excluded. No minimum duration of ARPI exposure was required. Patients who discontinued treatment within the first 3 months remained eligible, and progression or death occurring during this period was included from the date of ARPI initiation.

Because this was a retrospective fixed-cohort study, study size was determined by all eligible patients identified during the predefined study period, and no a priori sample-size calculation was performed. Penalized regression and bootstrap internal validation were used during model development to reduce overfitting and quantify optimism, while uncertainty in external-validation performance was reported using 95% confidence intervals (CIs).

### Clinical data and biomarker assessment

Baseline demographic, clinicopathological, treatment, and laboratory data were obtained from institutional electronic medical records. Variables included age, body mass index (BMI), smoking history, modified Charlson Comorbidity Index (mCCI),^18^ Eastern Cooperative Oncology Group (ECOG) performance status, International Society of Urological Pathology (ISUP) grade group, bone and visceral metastases, and metastatic disease volume according to the CHAARTED criteria. Baseline laboratory measurements, including complete blood count, alkaline phosphatase (ALP), lactate dehydrogenase (LDH), albumin, C-reactive protein (CRP), prostate-specific antigen (PSA), and 25(OH)D, were obtained within 4 weeks before ARPI initiation. The Nutritional C-Reactive Protein Ratio (NCR) was calculated as BMI (kg/m²) × serum albumin (g/L)/ CRP (mg/L).^19^

Serum 25(OH)D was measured using automated institutional immunoassays. The Beckman Coulter DxI 800 chemiluminescent immunoassay system was used at Etlik City Hospital and Gazi University, whereas the Roche Cobas e601 electrochemiluminescence immunoassay system was used at Kütahya City Hospital. Routine institutional quality-control procedures were applied. No centralized cross-platform calibration or mathematical harmonization was performed.

### Treatment exposure

Systemic treatment consisted of continuous ADT combined with abiraterone, enzalutamide, or apalutamide. Treatment was categorized as ADT plus an ARPI (doublet therapy) or ADT plus an ARPI plus docetaxel (triplet therapy). For patients receiving triplet therapy, docetaxel was planned at 75 mg/m² every 3 weeks for 6 cycles.

Treatment selection reflected contemporary institutional practice and treating-physician judgment based on disease volume, performance status, age, comorbidity burden, and fitness for docetaxel. The calendar year of ARPI initiation was recorded to account for temporal changes in treatment practice. Further details regarding docetaxel exposure and treatment-related covariates are provided in the Supplementary Methods.

### Clinical endpoints and follow-up

The primary endpoint was PFS, defined as the time from ARPI initiation to the first occurrence of documented objective clinical progression, radiographic progression according to Prostate Cancer Clinical Trials Working Group 3 criteria, or death from any cause. Events occurring within the first 3 months after ARPI initiation were retained, and no landmark or minimum-treatment-exposure restriction was applied.

OS was defined as the time from ARPI initiation to death from any cause. Patients without a documented PFS or OS event were censored at the date of last clinical follow-up. Follow-up continued until the relevant event, last clinical contact, or the study data cutoff in May 2026, as applicable.

### Statistical analysis

Reporting of prediction-model development and external validation followed the TRIPOD+AI statement.^20^ Continuous variables were summarized as median (IQR), and categorical variables as *n* (%). Between-cohort comparisons used the Mann–Whitney *U*, Pearson chi-square, or Fisher exact test, as appropriate. Right-skewed variables, including PSA, were natural-log transformed before regression modeling.

The primary analytic cohort included patients with available baseline 25(OH)D and sufficient baseline and follow-up data. Missing 25(OH)D values were not imputed. Potential selection from complete-case inclusion was assessed using standardized mean differences (SMDs) and an inverse-probability-of-inclusion weighted sensitivity analysis, as detailed in the Supplementary Methods.

PFS and OS were estimated using Kaplan–Meier methods, with log-rank comparisons; median follow-up was estimated using the reverse Kaplan–Meier method. A data-derived 25(OH)D cutoff identified by maximally selected rank statistics was used only for descriptive Kaplan–Meier visualization. In all regression-based prognostic analyses, 25(OH)D was modeled continuously, with HRs reported per 10-ng/mL increase.

For PFS model development, 10 candidate baseline predictors were specified before penalized fitting: age, ECOG performance status, ISUP grade group, CHAARTED disease volume, visceral metastasis, log-transformed PSA, hemoglobin, ALP, LDH, and treatment intensification. Post-treatment variables were excluded. Clinical predictor selection was performed only in the training cohort using LASSO-penalized Cox regression with standardized continuous predictors and 5-fold cross-validation; predictors with nonzero coefficients at *λ*min were retained. Baseline 25(OH)D was excluded from LASSO to permit evaluation of its incremental contribution. Model A included the LASSO-retained clinical predictors, whereas Model B added continuous 25(OH)D. Post-LASSO Cox models were fitted on the original predictor scales.

Model A and Model B were compared using Harrell’s C-index, ΔC-index, and likelihood-ratio testing. Internal validation used 1000 bootstrap resamples to estimate optimism-corrected discrimination. Calibration and time-dependent discrimination were assessed at 1 and 2 years, and decision-curve analysis (DCA) was exploratory. A 1- and 2-year PFS nomogram was derived from Model B. For external validation, training-derived coefficients and baseline survival functions were applied without predictor reselection, refitting, or recalibration; C-index, time-dependent AUC, calibration, and between-model differences in discrimination were evaluated.

Sensitivity analyses addressed complete-case selection, season, renal function, treatment heterogeneity, calendar year, and exploratory 25(OH)D-by-ARPI interactions. Proportional hazards, multicollinearity, and the functional form of 25(OH)D were also assessed. Because of the limited number of deaths, OS regression analyses were exploratory and were not used for model development. All tests were two-sided, with *P* < .05 considered statistically significant. Analyses used IBM SPSS Statistics version 25.0 and Python version 3.12; computational details are provided in the Supplementary Methods.

### Ethical considerations

The study was conducted in accordance with the Declaration of Helsinki and was approved by the Ankara Etlik City Hospital Scientific Research and Evaluation Ethics Committee (Approval No. AEŞH-BADEK2-2025-742). Because of the retrospective observational design, the requirement for written informed consent was waived by the institutional ethics committee. All clinical data were de-identified before analysis.

A separate publicly accessible study protocol was not prepared for this retrospective observational study, and the study was not prospectively registered. Patients and members of the public were not involved in the design, conduct, analysis, interpretation, or reporting of this retrospective study.

## Results

### Baseline patient characteristics

A total of 385 patients with *de novo* mHSPC initiating ARPI-based therapy were screened. After application of the eligibility criteria, 273 patients were included in the final analytic cohort. The training cohort comprised 192 patients treated at Etlik City Hospital, and the external validation cohort comprised 81 patients treated at Gazi University and Kütahya City Hospital (Figure 1).

**Figure 1.**
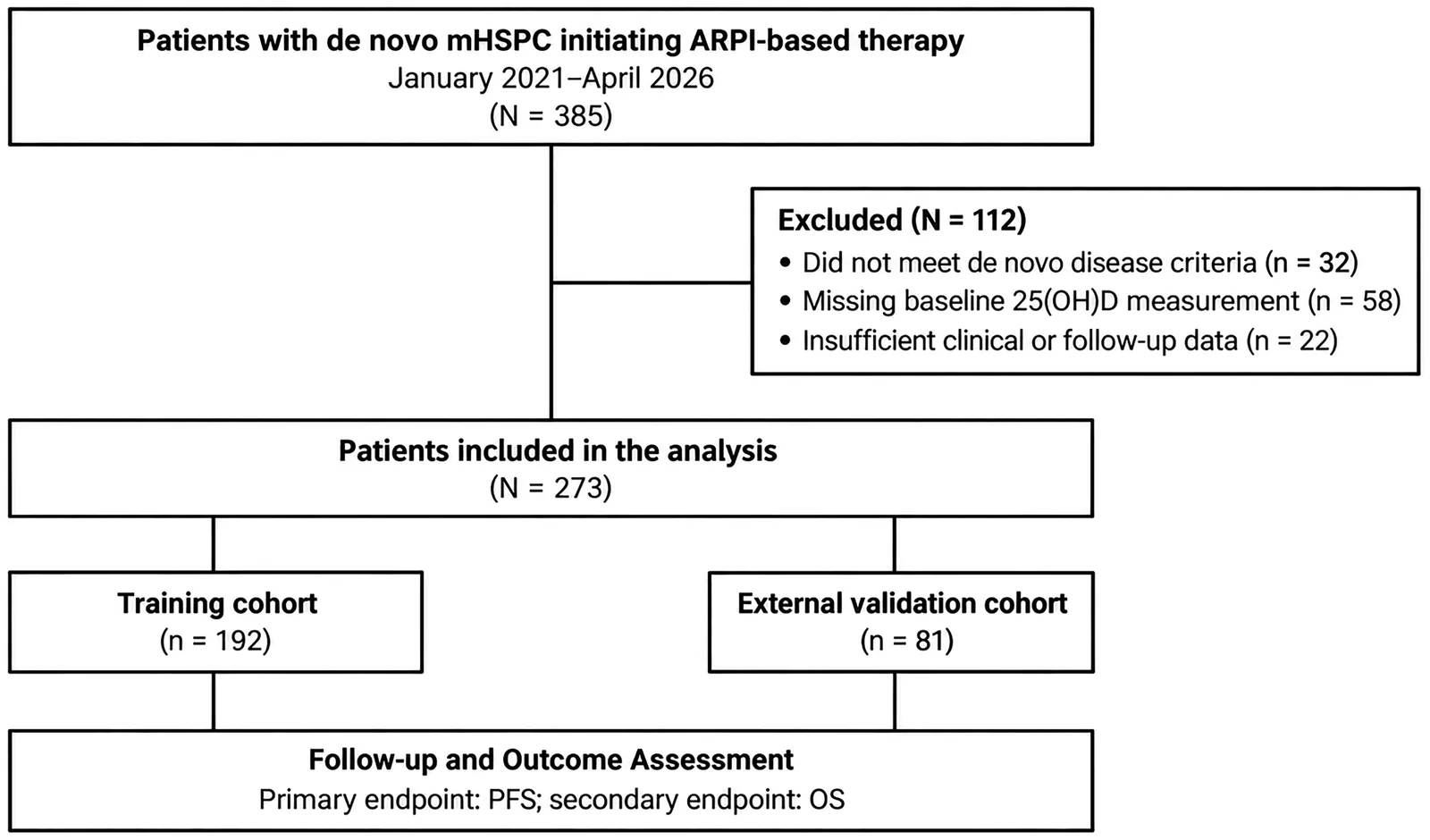
Flow chart of the study population selection. A total of 385 patients with *de novo* metastatic hormone-sensitive prostate cancer (mHSPC) treated with ARPI-based therapy between January 2021 and April 2026 across 3 tertiary centers were screened. Patients who did not meet the *de novo* disease criteria (*n* = 32), had missing baseline serum Vitamin D levels (*n* = 58), or had insufficient clinical or follow-up data (*n* = 22) were excluded. A total of 273 patients were included in the final analysis and stratified into training (*n* = 192) and external validation (*n* = 81) cohorts. Follow-up and outcome assessment were performed to evaluate the primary endpoint, progression-free survival (PFS), and the secondary endpoint, overall survival (OS). No patient was excluded on the basis of ARPI treatment duration, and no minimum ARPI exposure requirement was applied for study eligibility. Abbreviations: ARPI, androgen receptor pathway inhibitor; mHSPC, metastatic hormone-sensitive prostate cancer; PFS, progression-free survival.

The median age in the overall cohort was 70.0 years (IQR, 64.0-77.0), and 200 patients (73.3%) were aged 65 years or older. The median BMI was 26.0 kg/m² (IQR, 23.9-28.2), and the median baseline 25(OH)D concentration was 20.0 ng/mL (IQR, 11.0-27.0). High-volume disease according to the CHAARTED criteria was present in 168 patients (61.5%), and 210 (76.9%) had bone metastases. Overall, 228 patients (83.5%) received ADT plus an ARPI and 45 (16.5%) received triplet therapy. Baseline characteristics of the training and external validation cohorts are presented in Table 1. Baseline demographic, disease-related, laboratory, and treatment characteristics according to the 19.0-ng/mL 25(OH)D threshold are presented in Table S1.

**Table 1.** Baseline demographic and clinicopathological characteristics of the training and validation cohorts.

Characteristics (*n* = 273)	Total cohort (*n* = 273)	Training cohort (*n* = 192)	Validation cohort (*n* = 81)	*P*-value
**Age (years), median (IQR)**	70.0 (64.0-77.0)	70.0 (64.0-77.0)	70.0 (65.0-75.0)	.333
**Age group, *n* (%)**				.656
** <65**	73 (26.7)	53 (27.6)	20 (24.7)	
** ≥65**	200 (73.3)	139 (72.4)	61 (75.3)	
**Body mass index (kg/m²), median (IQR)**	26.0 (23.9-28.2)	26.2 (24.2-28.2)	25.9 (23.5-27.5)	.179
**mCCI, median (IQR)**	9.0 (8.0-11.0)	10.0 (8.0-11.0)	9.0 (8.0-10.0)	.175
**mCCI score category, *n* (%)**				.296
** <8**	47 (17.2)	30 (15.6)	17 (21.0)	
** ≥8**	226 (82.8)	162 (84.4)	64 (79.0)	
**Smoking history, *n* (%)**				.660
** Absent**	78 (28.6)	53 (27.6)	25 (30.9)	
** Present**	195 (71.4)	139 (72.4)	56 (69.1)	
**ECOG performance status, *n* (%)**				.520
** 0-1**	214 (78.4)	148 (77.1)	66 (81.5)	
** ≥2**	59 (21.6)	44 (22.9)	15 (18.5)	
**ISUP risk category, *n* (%)**				.310
** Low/intermediate (grade 1-3)**	81 (29.7)	61 (31.8)	20 (24.7)	
** High (grade 4-5)**	192 (70.3)	131 (68.2)	61 (75.3)	
**Disease volume (CHAARTED criteria), *n* (%)**				.104
** Low volume**	105 (38.5)	80 (41.7)	25 (30.9)	
** High volume**	168 (61.5)	112 (58.3)	56 (69.1)	
**Metastatic sites, *n* (%)**				
** Bone metastasis present**	210 (76.9)	144 (75.0)	66 (81.5)	.274
** Visceral metastasis present**	77 (28.2)	51 (26.6)	26 (32.1)	.379
**Vitamin D level, *n* (%)**				.865
** ≤19.0 ng/mL**	134 (49.1)	98 (51.0)	36 (44.4)	
** >19.0 ng/mL**	139 (50.9)	94 (49.0)	45 (55.6)	
**PSMA PET SUVmax, median (IQR)**	14.0 (8.0-23.0)	15.0 (8.0-23.0)	12.0 (8.0-22.0)	.365
**Serum 25(OH)D (ng/mL), median (IQR)**	20.0 (11.0-27.0)	19.0 (11.0-27.0)	23.0 (12.0-29.0)	.068
**NCR score, median (IQR)**	111.4 (62.6-216.2)	108.1 (60.6-199.1)	116.7 (65.4-227.2)	.767
**Baseline PSA (ng/mL), median (IQR)**	46.0 (10.0-148.0)	46.0 (9.0-143.0)	50.0 (14.0-162.0)	.372
**Baseline laboratory parameters, median (IQR)**				
**Hemoglobin (g/dL)**	13.7 (12.4-14.6)	13.7 (12.4-14.4)	13.7 (12.4-14.7)	.568
**White blood cell count (×10³/µL)**	7.2 (6.2-8.5)	7.2 (6.0-8.2)	7.3 (6.5-9.2)	.092
**Absolute neutrophil count (×10³/µL)**	4.4 (3.6-5.0)	4.4 (3.6-4.9)	4.2 (3.6-5.3)	.611
**Absolute lymphocyte count (×10³/µL)**	2.0 (1.4-2.3)	1.9 (1.4-2.3)	2.1 (1.6-2.6)	.035
**Platelet count (×10³/µL)**	238.0 (201.0-288.0)	239.0 (203.2-286.0)	228.0 (194.0-301.0)	.826
**Neutrophil-to-lymphocyte ratio (NLR)**	2.1 (1.8-3.1)	2.1 (1.8-3.1)	2.0 (1.7-2.8)	.143
**Serum albumin (g/L)**	44.0 (41.0-45.0)	44.0 (40.8-45.0)	44.0 (41.0-46.0)	.210
**C-reactive protein (mg/L)**	9.8 (5.5-17.0)	9.8 (5.6-16.9)	9.3 (4.9-17.1)	.726
**Lactate dehydrogenase (U/L)**	189.0 (170.0-222.0)	188.0 (168.8-224.2)	190.0 (173.0-211.0)	.824
**Alkaline phosphatase (U/L)**	97.0 (78.0-142.0)	95.0 (78.8-128.0)	100.0 (77.0-155.0)	.419
**Systemic treatment characteristics, *n* (%)**				
** ADT type**				.829
** LHRH agonist**	225 (82.4)	160 (83.3)	65 (80.2)	
** LHRH antagonist**	33 (12.1)	22 (11.5)	11 (13.6)	
** Bilateral orchiectomy**	15 (5.5)	10 (5.2)	5 (6.2)	
** ARPI agent type**				.162
** Enzalutamide**	134 (49.1)	90 (46.9)	44 (54.3)	
** Abiraterone**	121 (44.3)	86 (44.8)	35 (43.2)	
** Apalutamide**	18 (6.6)	16 (8.3)	2 (2.5)	
** Treatment intensification**				.723
** ADT + ARPI**	228 (83.5)	159 (82.8)	69 (85.2)	
** Triplet**	45 (16.5)	33 (17.2)	12 (14.8)	

Abbreviations: 25(OH)D, 25-hydroxyvitamin D; ADT, androgen deprivation therapy; ARPI, androgen receptor pathway inhibitor; ECOG, Eastern Cooperative Oncology Group; ISUP, International Society of Urological Pathology; LHRH, luteinizing hormone-releasing hormone; mCCI, modified Charlson Comorbidity Index; NCR, nutritional C-reactive protein ratio; NLR, neutrophil-to-lymphocyte ratio; PET, positron emission tomography; PSA, prostate-specific antigen; PSMA, prostate-specific membrane antigen; SUVmax, maximum standardized uptake value.

Data are presented as median (interquartile range) for continuous variables and number (percentage) for categorical variables. P-values represent comparisons between the training and external validation cohorts and were calculated using the Mann-Whitney U test for continuous variables and the Pearson chi-square or Fisher exact test for categorical variables, as appropriate.

### Assessment of complete-case selection

Among 353 patients who otherwise met the disease-related eligibility criteria, 273 were included in the analytic cohort and 80 were excluded because baseline 25(OH)D measurements were unavailable (*n* = 58) or clinical or follow-up data were insufficient (*n* = 22). The maximum absolute SMD across the evaluated demographic, disease-related, laboratory, and treatment characteristics was 0.15. Available-case 1-year PFS was 88.1% in the included cohort and 85.9% in the excluded cohort (*P* = .460), and death during available follow-up occurred in 13.5% and 15.5%, respectively (*P* = .680; Table S2).

### Follow-up and survival outcomes

Median follow-up estimated using the reverse Kaplan–Meier method was 23.5 months (95% CI, 21.3-25.6) in the overall cohort, 25.5 months (95% CI, 21.7-28.4) in the training cohort, and 20.8 months (95% CI, 18.6-23.1) in the external validation cohort.

During follow-up, 112 patients (41.0%) experienced a PFS event and 37 (13.5%) died. Median PFS in the overall cohort was 35.0 months (95% CI, 28.1-39.5), with 1- and 2-year PFS rates of 88.1% (95% CI, 83.6-91.4) and 70.0% (95% CI, 63.5-75.6), respectively. Median OS was 54.5 months (95% CI, 50.0-not reached [NR]), with 1- and 2-year OS rates of 97.0% (95% CI, 94.1-98.5) and 91.9% (95% CI, 87.1-95.0), respectively.

In the training cohort, 85 patients experienced a PFS event and 27 died. Median PFS was 35.0 months (95% CI, 27.1-37.0), and median OS was 56.6 months (95% CI, 51.0-NR). In the external validation cohort, 27 patients experienced a PFS event and 10 died. Median PFS was 34.7 months (95% CI, 27.2-43.1), and median OS was 52.0 months (95% CI, 41.3-NR).

### Baseline 25(OH)D and survival stratification

Maximally selected rank statistics identified a data-derived baseline 25(OH)D threshold of 19.0 ng/mL, which was used for descriptive Kaplan–Meier analyses. In the training cohort, median PFS was 21.1 months (95% CI, 18.2-27.1) in the ≤19.0-ng/mL group and was not reached (NR) (95% CI, 45.4-NR) in the > 19.0-ng/mL group (log-rank *P* < .001; Figure 2A). Median OS was 54.5 months (95% CI, 50.0-NR) and NR (95% CI, 50.0-NR), respectively (log-rank *P* = .033; Figure 2B).

**Figure 2.**
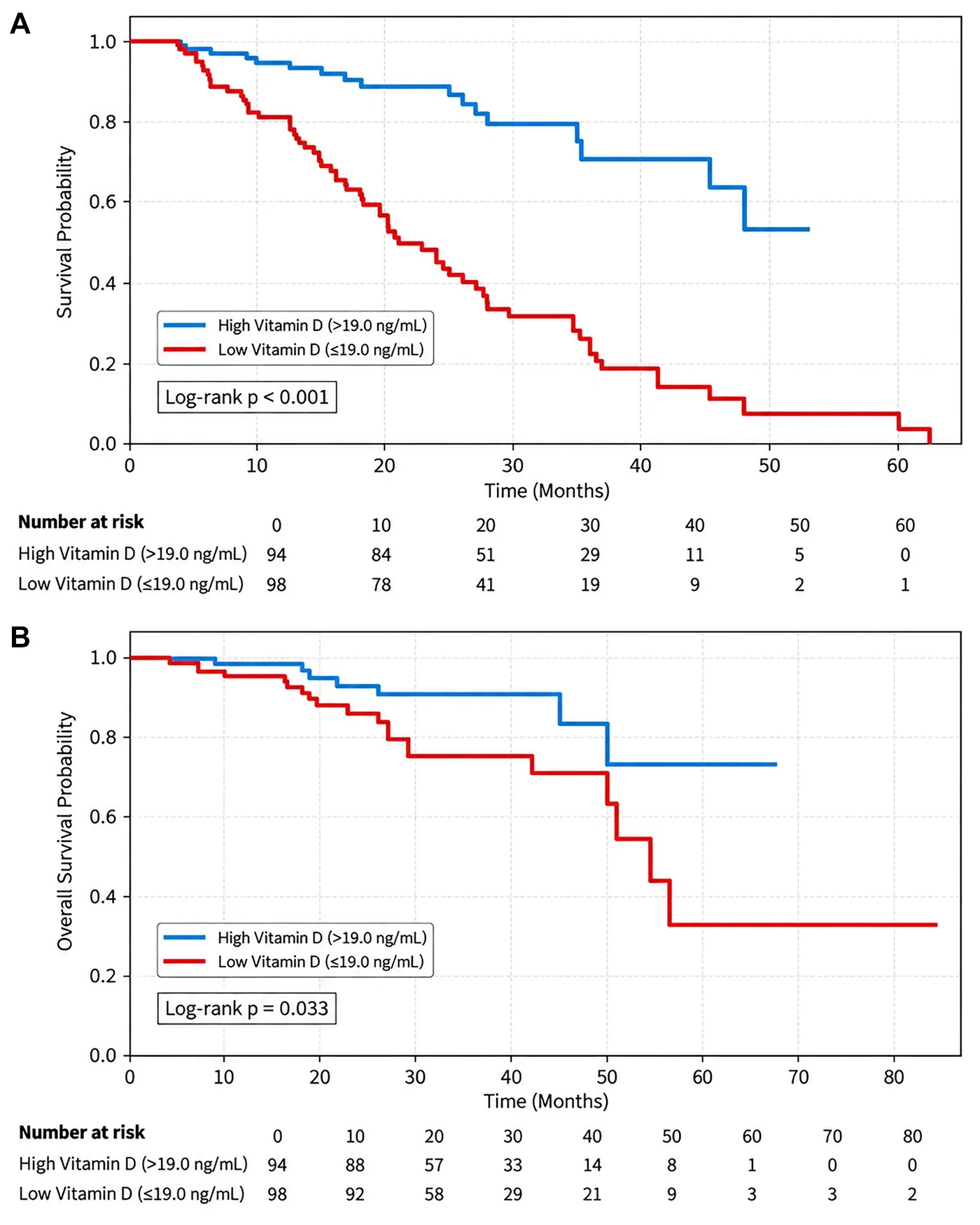
Kaplan-Meier survival curves for (A) progression-free survival (PFS) and (B) overall survival (OS) stratified by baseline serum Vitamin D levels in the training cohort. A baseline serum 25(OH)D cutoff of 19.0 ng/mL, identified using maximally selected rank statistics, was used for Kaplan-Meier visualization. In the training cohort, median PFS was 21.1 months (95% CI, 18.2-27.1) in the low 25(OH)D group (≤19.0 ng/mL), whereas median PFS was not reached (95% CI, 45.4-NR) in the high 25(OH)D group (>19.0 ng/mL; log-rank *P* < .001). Median OS was 54.5 months (95% CI, 50.0-NR) in the low 25(OH)D group and was not reached (95% CI, 50.0-NR) in the high 25(OH)D group (log-rank *P* = .033). Abbreviations: NR, not reached; PFS, progression-free survival.

When the same threshold was applied unchanged to the external validation cohort, median PFS was 34.7 months (95% CI, 27.2-39.6) in the ≤19.0-ng/mL group and was NR (95% CI, 25.0-NR) in the >19.0-ng/mL group (log-rank *P* = .018; Figure S1A, see online supplementary material for a color version of this figure). Median OS was 49.9 months (95% CI, 41.3-52.5) and NR (95% CI, 28.7-NR), respectively (log-rank *P* = .042; Figure S1B, see online supplementary material for a color version of this figure).

### Prognostic model development and incremental value of baseline 25(OH)D

LASSO-penalized Cox regression retained age, ISUP grade group, CHAARTED disease volume, baseline log-transformed PSA, and hemoglobin from the 10 prespecified candidate baseline predictors in the training cohort. These variables comprised Model A (Table S3). Model B comprised the same clinical predictor set with the addition of continuous baseline 25(OH)D.

In Model A, age (HR, 1.045 per year; 95% CI, 1.019-1.072; *P* < .001), high CHAARTED disease volume (HR, 1.720; 95% CI, 1.042-2.838; *P* = .034), and baseline log-transformed PSA (HR, 1.223 per 1-unit increase; 95% CI, 1.091-1.372; *P* < .001) were associated with PFS. In Model B, age (HR, 1.041 per year; 95% CI, 1.015-1.068; *P* = .002), baseline log-transformed PSA (HR, 1.173 per 1-unit increase; 95% CI, 1.047-1.315; *P* = .006), and baseline 25(OH)D (HR, 0.635 per 10-ng/mL increase; 95% CI, 0.488-0.825; *P* < .001) were associated with PFS (Table 2).

**Table 2.** Post-LASSO multivariable cox models for progression-free survival in the training cohort.

Predictor	Model A: clinical model HR (95% CI)	*P*-value	Model B: clinical + 25(OH)D HR (95% CI)	*P*-value
**Age, per 1-year increase**	1.045 (1.019-1.072)	<.001	1.041 (1.015-1.068)	.002
**ISUP grade 4-5 vs 1-3**	1.378 (0.747-2.542)	.305	1.199 (0.651-2.209)	.560
**CHAARTED disease volume, high vs low**	1.720 (1.042-2.838)	.034	1.606 (0.970-2.658)	.065
**Baseline log-PSA, per 1-unit increase**	1.223 (1.091-1.372)	<.001	1.173 (1.047-1.315)	.006
**Hemoglobin, per 1-g/dL increase**	0.924 (0.810-1.055)	.243	0.961 (0.840-1.099)	.561
**Baseline 25(OH)D, per 10-ng/mL increase**	—	—	0.635 (0.488-0.825)	<.001

Abbreviations: 25(OH)D, 25-hydroxyvitamin D; HR, hazard ratio; ISUP, International Society of Urological Pathology; PSA, prostate-specific antigen.

Model A comprised the clinical predictors retained by LASSO-penalized Cox regression using 5-fold cross-validation, with the penalty parameter selected at the value minimizing the cross-validated partial-likelihood deviance (λmin). Model B comprised the identical clinical predictor set plus baseline 25(OH)D, modeled continuously per 10-ng/mL increase. Post-LASSO Cox regression estimates are presented on the original predictor scales. Predictors retained by LASSO were preserved in both models irrespective of their individual *P*-values in the post-LASSO Cox models. Both models were fitted in the training cohort (*n* = 192; 85 progression-free survival events).

The apparent Harrell C-index was 0.712 (95% CI, 0.654-0.783) for Model A and 0.740 (95% CI, 0.684-0.809) for Model B. The difference in C-index was +0.028 (95% CI, −0.003 to 0.060). The likelihood-ratio test comparing the nested models yielded χ² = 12.71 (*P* < .001). Mean bootstrap optimism was 0.015 for Model A and 0.018 for Model B. The corresponding optimism-corrected C-indices were 0.697 (95% CI, 0.638-0.764) and 0.722 (95% CI, 0.662-0.784), respectively (Table 3).

**Table 3.** Model performance and incremental prognostic value for progression-free survival in the training cohort.

Performance measure	Model A: clinical model	Model B: clinical + 25(OH)D	Increment/comparison
**Apparent Harrell C-index (95% CI)**	0.712 (0.654-0.783)	0.740 (0.684-0.809)	ΔC-index = +0.028 (95% CI, −0.003 to 0.060)
**Mean bootstrap optimism**	0.015	0.018	—
**Optimism-corrected C-index (95% CI)**	0.697 (0.638-0.764)	0.722 (0.662-0.784)	—
**Likelihood-ratio test**	—	—	χ² = 12.71; *P* < .001

Abbreviation: 25(OH)D, 25-hydroxyvitamin D.

Harrell C-index confidence intervals and the 95% CI for ΔC-index were estimated using paired bootstrap resampling. Internal validation of the final post-LASSO models was performed using 1000 bootstrap resamples to estimate optimism and derive optimism-corrected C-indices. Bootstrap 95% CIs for the optimism-corrected C-indices were derived from the distribution of iteration-specific optimism-corrected performance estimates.

### Nomogram and external validation

A nomogram based on Model B was constructed to estimate 1- and 2-year PFS probabilities (Figure 3).

**Figure 3.**
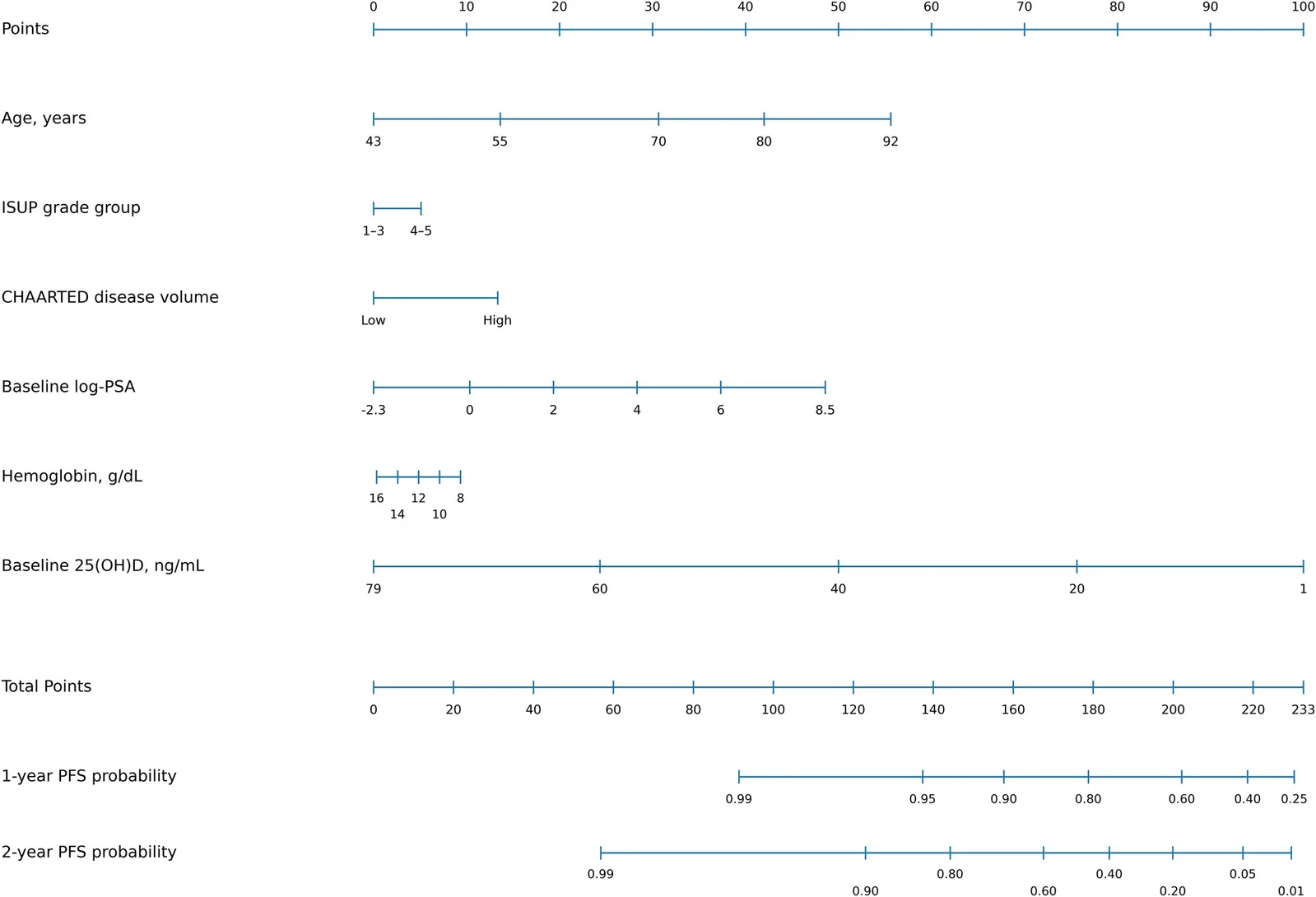
Nomogram for 1- and 2-year progression-free survival based on the clinical-plus-25(OH)D Model. The nomogram was constructed from Model B in the training cohort using the post-LASSO Cox regression coefficients. Model B included age, ISUP grade group, CHAARTED disease volume, baseline log-transformed PSA, hemoglobin, and baseline 25(OH)D. For each predictor, the corresponding value is mapped to the points scale, and the individual points are summed to obtain the total points. Total points are then mapped to the estimated 1- and 2-year PFS probabilities. Higher total points correspond to lower predicted PFS probabilities. Baseline 25(OH)D was modeled continuously, and the 25(OH)D axis is displayed in ng/mL. Complete Model B coefficients, baseline survival estimates, and instructions for calculation of individualized PFS probabilities are provided in Table S5. Abbreviations: 25(OH)D, 25-hydroxyvitamin D; ISUP, International Society of Urological Pathology; PFS, progression-free survival; PSA, prostate-specific antigen.

In the external validation cohort, training-derived coefficients were applied without refitting or recalibration. The Harrell C-index was 0.71 (95% CI, 0.64-0.78) for Model A and 0.76 (95% CI, 0.69-0.83) for Model B, with a difference of +0.05 (95% CI, 0.01-0.10). The corresponding 1-year AUCs were 0.72 and 0.78, and the 2-year AUCs were 0.74 and 0.80, with between-model differences of +0.06 at both time points (95% CI, 0.01-0.12; Table S4). Complete Model B coefficients, baseline survival estimates, and calculation details are provided in Table S5.

Time-dependent ROC curves are presented in Figure S2 (see online supplementary material for a color version of this figure), with calibration analyses for the training and external validation cohorts in Figures S3 and S4 (see online supplementary material for a color version of these figures), respectively. Exploratory DCA are shown in Figure S5 (see online supplementary material for a color version of this figure).

### Sensitivity and exploratory analyses

Sensitivity analyses are summarized in Table S6. The HR per 10-ng/mL increase in baseline 25(OH)D was 0.65 (95% CI, 0.50-0.85; *P* = .002) after inverse-probability-of-inclusion weighting, 0.66 (95% CI, 0.51-0.86; *P* = .002) in the prespecified clinical model, and 0.68 (95% CI, 0.52-0.88; *P* = .007) after simultaneous adjustment for season and baseline eGFR.

Among 45 patients receiving triplet therapy, all received docetaxel at 75 mg/m² every 3 weeks for 6 planned cycles; 41 (91.1%) completed all 6 cycles, and 42 (93.3%) initiated docetaxel within 30 days of ARPI initiation. Additional treatment characteristics and dose modifications are reported in Table S7. In the model adjusted for ARPI agent, treatment intensification, and calendar year, the HR for baseline 25(OH)D was 0.65 (95% CI, 0.49-0.85; *P* = .002).

In exploratory ARPI-specific analyses, the HR per 10-ng/mL increase in 25(OH)D was 0.59 (95% CI, 0.39-0.89) for enzalutamide, 0.72 (95% CI, 0.51-1.01) for abiraterone, and 0.30 (95% CI, 0.10-0.92) for apalutamide. The global interaction test yielded *P* = .313 (Table S7).

RCS analysis yielded *P* = .001 for the overall association and *P* = .327 for nonlinearity (Figure S6, see online supplementary material for a color version of this figure). In the exploratory multivariable OS analysis, the HR for baseline 25(OH)D was 0.651 per 10-ng/mL increase (95% CI, 0.420-0.991; *P* = .048; Table S8). Post-treatment PSA characteristics are reported descriptively in Table S9 and were not used for baseline model development.

## Discussion

In this multicenter retrospective study of patients with *de novo* mHSPC initiating contemporary ARPI-based therapy, higher baseline serum 25(OH)D was associated with longer PFS and provided prognostic information beyond a clinical model derived using penalized predictor selection. Specifically, each 10-ng/mL increase in baseline 25(OH)D was associated with an approximately 36% lower hazard of progression, and addition of 25(OH)D improved model fit while producing a modest increase in discrimination. The association remained consistent across multiple sensitivity analyses and was reproduced in an independent external validation cohort, whereas OS findings remained exploratory because of the limited number of deaths. Collectively, these findings support baseline 25(OH)D as a complementary prognostic marker for risk stratification in *de novo* mHSPC rather than as a predictive biomarker or a basis for treatment selection.

The association between baseline 25(OH)D and prognosis is biologically plausible and consistent with prior observational evidence in PC. Vitamin D receptor signaling has been implicated in the regulation of cellular proliferation, apoptosis, inflammatory pathways, Wnt/β-catenin signaling, and androgen receptor-related mechanisms.^8^^,^^9^^,^^21–23^ Clinically, lower circulating 25(OH)D concentrations have been associated with adverse pathological features and poorer PC outcomes in observational studies and meta-analyses.^10–13^ However, much of this evidence derives from localized disease or heterogeneous PC populations, and data specifically addressing *de novo* mHSPC treated with contemporary ARPI-based therapy remain limited. Importantly, observational prognostic associations should not be interpreted as evidence that vitamin D supplementation improves cancer outcomes. Systematic reviews and clinical trials have yielded inconsistent evidence regarding supplementation,^14^^,^^15^ and the recent ProsD randomized phase II trial did not demonstrate a significant reduction in disease progression with high-dose vitamin D supplementation among patients with localized low- to intermediate-risk PC managed with active surveillance.^16^ Although that clinical setting differs substantially from *de novo* metastatic disease, the ProsD findings reinforce the distinction between prognostic association and therapeutic benefit. Therefore, our findings should be interpreted as supporting the prognostic relevance of baseline 25(OH)D rather than a direct antitumor effect or a rationale for vitamin D supplementation as cancer therapy.

Our study extends prior work on host-related prognostic biomarkers in mHSPC by evaluating baseline 25(OH)D within a contemporary ARPI-treated population and by testing whether this biomarker adds information beyond established clinical factors. Previous studies have shown that nutritional and inflammatory indices, particularly the prognostic nutritional index, may identify patients with mHSPC at higher risk of adverse outcomes,^24^^,^^25^ and a recent meta-analysis further supported the prognostic relevance of nutritional status across PC populations receiving ADT.^26^ However, these studies primarily evaluated composite inflammatory or nutritional markers, included heterogeneous treatment settings or disease presentations, and generally focused on prognostic association rather than the incremental contribution of a new biomarker to an existing clinical model. In contrast, the present study focused exclusively on *de novo* mHSPC treated with contemporary ARPI-based therapy, evaluated 25(OH)D as a continuous baseline biomarker, and incorporated independent external validation without model refitting. These features strengthen the evidence that 25(OH)D may provide complementary prognostic information rather than simply reproducing established measures of disease burden or host vulnerability. Nevertheless, because our study remains observational and the external validation cohort was relatively small, these findings should be regarded as an extension of current prognostic research rather than evidence that 25(OH)D is superior to existing biomarkers or ready for routine clinical implementation.

The consistency of the 25(OH)D association across treatment-related and sensitivity analyses also provides important context for interpreting its potential role in contemporary mHSPC. Randomized trials have established substantial survival benefits from ARPI-based treatment intensification, but treatment selection in routine practice still relies predominantly on clinical factors such as disease burden, fitness, comorbidity, and expected treatment tolerance.^2–6^^,^^27^ At the same time, recent biomarker research has highlighted the need for biologically informed tools that complement conventional clinicopathological stratification, although few candidate biomarkers have reached a level of evidence sufficient to guide treatment selection in metastatic hormone-sensitive disease.^28^ In our study, the prognostic association of baseline 25(OH)D was not materially altered after accounting for treatment strategy or other clinically relevant sources of variation, suggesting that the observed signal may not simply reflect the choice of ARPI or treatment intensification. However, the absence of a statistically detectable interaction across ARPI agents should not be interpreted as evidence of equivalent effects, particularly given the limited size of treatment-specific subgroups. This distinction is especially relevant for abiraterone, because prednisone forms an integral component of that regimen and its potential contribution to vitamin D metabolism could not be separated from the effect of abiraterone itself. Similarly, although the descriptive 25(OH)D threshold and DCA provide supportive evidence for risk stratification, neither establishes a treatment threshold or demonstrates that 25(OH)D should influence ARPI selection, triplet intensification, or surveillance. Thus, our findings extend current biomarker research by identifying a readily available candidate prognostic marker that appears robust across contemporary treatment contexts, while prospective studies with adequately powered treatment-by-biomarker interaction analyses will be required before any treatment-predictive role can be considered.

Several strengths of the present study should be acknowledged. The study evaluated a clinically well-defined cohort of patients with *de novo* mHSPC treated with contemporary ARPI-based therapy and incorporated an independent multicenter external validation cohort. Baseline 25(OH)D was evaluated primarily as a continuous variable, thereby reducing dependence on an arbitrary deficiency threshold, and the prognostic model was developed using penalized predictor selection with internal bootstrap validation. The findings were further examined across multiple sensitivity analyses, including analyses addressing selection, season of measurement, renal function, and treatment-related factors, while restricted cubic spline modeling supported the use of 25(OH)D as a continuous predictor. Together, these features strengthen the methodological rigor and reproducibility of the observed prognostic association. Several limitations should also be considered. First, the retrospective observational design introduces the possibility of residual confounding and selection bias. Patients without an available baseline 25(OH)D measurement could not contribute to the primary biomarker analysis; although comparisons between included and excluded patients and inverse-probability-of-inclusion weighting provided some reassurance against major observed selection effects, unmeasured determinants of vitamin D testing, data completeness, or clinical follow-up may still have influenced the findings. Second, although season of blood collection and renal function were addressed in sensitivity analyses, information on vitamin D supplementation, including post-baseline initiation, dose, formulation, and timing, as well as sunlight exposure, dietary intake, and potentially relevant concomitant medications was not consistently available. In addition, 25(OH)D was measured using different institutional immunoassay platforms without centralized cross-platform calibration, introducing potential measurement heterogeneity. Third, 25(OH)D was assessed at a single baseline time point, precluding evaluation of whether longitudinal changes in vitamin D status paralleled treatment response, nutritional recovery, or changes in systemic inflammation. Fourth, treatment allocation was nonrandom, and residual confounding related to ARPI selection or treatment intensification cannot be excluded despite treatment-adjusted sensitivity analyses; similarly, the contribution of concomitant prednisone could not be separated from abiraterone-based therapy. Fifth, the external validation cohort was relatively small, and the number of outcome events, particularly deaths, was limited, reducing the precision of model performance and OS estimates. Race or ethnicity and socioeconomic data were not consistently available, precluding assessment of model fairness and transportability across these sociodemographic groups. Finally, the study was not designed to establish biological mechanism, causality, therapeutic benefit from vitamin D supplementation, or clinical benefit from using 25(OH)D-guided risk estimates to alter treatment. Therefore, the findings should be interpreted as evidence of a prognostic association and incremental prognostic information that requires prospective validation, rather than proof that correcting vitamin D deficiency or implementing the model improves patient outcomes.

## Conclusion

In conclusion, baseline serum 25(OH)D was independently associated with PFS in patients with *de novo* mHSPC receiving contemporary ARPI-based therapy, whereas the OS findings remained exploratory. Adding baseline 25(OH)D to a clinical prognostic model provided incremental prognostic information, with supportive performance in an independent external validation cohort. These findings support further evaluation of baseline 25(OH)D as a readily accessible candidate prognostic biomarker in contemporary mHSPC. However, the present study does not establish a predictive role for 25(OH)D or demonstrate therapeutic benefit from vitamin D supplementation. Prospective multicenter studies should confirm model transportability and evaluate clinician-assisted use before implementation; predictions should not be generated when required baseline inputs are unavailable.

## Supplementary Material

oyag396_Supplementary_Data

## Data Availability

The datasets generated and/or analyzed during the current study are not publicly available due to institutional data protection regulations but are available from the corresponding author upon reasonable request. The analytical code used for model development and evaluation is available from the corresponding author upon reasonable request.
