# Supplementary material for: Baseline 25-hydroxyvitamin D and progression-free survival in de novo metastatic hormone-sensitive prostate cancer: prognostic model development and external validation
Source: Oncologist. 2026 Oct 6;31(11):oyag396. doi: 10.1093/oncolo/oyag396 (PMC13680101; doi:10.1093/oncolo/oyag396)
Supplement: Supplementary file 1 [file oyag396_supplementary_data.docx]

**Supplementary Methods**

**Data Preparation, Predictor Assessment, and Outcome Ascertainment**

Data from the participating centers were harmonized using common variable definitions and coding rules before analysis. Data were reviewed for completeness, duplicate records, coding consistency, and clinically implausible values, with discrepancies checked against the source electronic medical records. The same preprocessing rules were applied across centers, and no sociodemographic-group-specific preprocessing was performed.

Candidate predictors represented information available at or before initiation of ARPI-based therapy. Age and ECOG performance status were defined at treatment initiation; ISUP grade group was obtained from diagnostic histopathology; metastatic sites and CHAARTED disease volume were derived from baseline staging assessments; and laboratory predictors were obtained within 4 weeks before ARPI initiation. All predictors therefore preceded subsequent PFS outcome ascertainment.

PFS events were ascertained retrospectively from routine clinical records and radiologic assessments. Radiographic progression was classified according to PCWG3 criteria, whereas documented clinical progression was obtained from treating-oncologist records. Assessment timing followed routine clinical practice rather than a study-mandated imaging schedule. No study-specific centralized or blinded outcome adjudication was performed. Similarly, predictor measurements and clinical classifications were obtained from routine-care assessments rather than by study-specific blinded assessors. Demographic characteristics of the clinical, radiologic, and pathological assessors were not collected.

**Complete-Case Selection and Inclusion Weighting**

The primary analysis included patients with an available baseline serum 25-hydroxyvitamin D [25(OH)D] measurement and sufficient baseline clinical and follow-up data. Missing baseline 25(OH)D values were not imputed because 25(OH)D was the primary biomarker of interest and was completely unobserved when the assay had not been performed.

To assess potential selection associated with complete-case inclusion, baseline demographic, disease-related, laboratory, and treatment characteristics were compared between patients included in the analytic cohort and otherwise eligible patients excluded because of unavailable baseline 25(OH)D measurements or insufficient clinical or follow-up data. Standardized mean differences (SMDs) were calculated to quantify between-group differences.

Among otherwise eligible patients, the probability of inclusion in the analytic cohort was estimated using multivariable logistic regression incorporating center, age, Eastern Cooperative Oncology Group (ECOG) performance status, International Society of Urological Pathology (ISUP) grade group, CHAARTED disease volume, visceral metastasis, baseline log-transformed prostate-specific antigen (PSA), androgen receptor pathway inhibitor (ARPI) agent, treatment intensification strategy, and calendar year of ARPI initiation. Stabilized inverse-probability-of-inclusion weights were derived from this model. The Model B covariate specification was subsequently evaluated using weighted Cox regression with robust variance.

Among the 273 patients in the analytic cohort, data required for the candidate predictors used in model development were complete; therefore, no additional predictor imputation was performed.

**Study Size Considerations**

Because this was a retrospective fixed-cohort study, no a priori sample-size calculation was performed. All eligible patients identified during the predefined study period were considered. The training cohort included 192 patients with 85 PFS events and 10 prespecified candidate predictors, whereas the external validation cohort included 81 patients with 27 PFS events. Penalized predictor selection and bootstrap internal validation were used to limit overfitting during model development. Given the limited number of events in the external validation cohort, performance estimates were reported with confidence intervals and interpreted cautiously.

**Penalized Predictor Selection and Model Development**

Candidate predictors for progression-free survival (PFS) were age, ECOG performance status, ISUP grade group, CHAARTED disease volume, visceral metastasis, baseline log-transformed PSA, hemoglobin, alkaline phosphatase, lactate dehydrogenase, and treatment intensification strategy. Continuous predictors were standardized before penalized fitting.

Least absolute shrinkage and selection operator (LASSO)-penalized Cox regression was performed exclusively in the training cohort. The penalty parameter was selected using 5-fold cross-validation based on partial-likelihood deviance, with λmin defined as the value minimizing the cross-validated deviance. Predictors with nonzero coefficients at λmin were retained. No predictor was selected or excluded according to univariable statistical significance.

Baseline 25(OH)D was intentionally excluded from penalized clinical predictor selection because the primary modeling objective was to evaluate its incremental prognostic contribution. Model A comprised the clinical predictors retained by LASSO, whereas Model B comprised the same clinical predictor set plus baseline 25(OH)D modeled continuously per 10-ng/mL increase. Post-LASSO Cox models were fitted on the original predictor scales, and all predictors retained by LASSO were preserved irrespective of their individual p-values.

**Internal Validation and Model Performance**

Discrimination was quantified using Harrell’s concordance index (C-index). The difference in C-index between Model A and Model B was estimated using paired bootstrap resampling with 95% confidence intervals (CIs). Improvement in model fit after addition of 25(OH)D was assessed using the likelihood-ratio test for nested models.

Internal validation was performed using 1,000 bootstrap resamples. Model A and Model B were refitted within each bootstrap sample to estimate optimism in Harrell’s C-index and derive optimism-corrected discrimination estimates.

Calibration was assessed at 1 and 2 years by comparing model-predicted PFS probabilities with Kaplan–Meier estimates of observed PFS. Time-dependent receiver operating characteristic analyses were performed at the same time points. Decision curve analysis was undertaken exploratorily to compare the net benefit of Model A and Model B. Because no specific treatment or surveillance action was prespecified for any probability threshold, decision curve analysis was not used to define a clinical decision threshold.

**Nomogram and External Validation**

A prognostic nomogram for 1- and 2-year PFS was constructed using the regression coefficients from Model B. The 1- and 2-year prediction horizons were selected because they represented clinically relevant early- and intermediate-term outcomes and were supported by the available follow-up and numbers at risk.

For external validation, the regression coefficients and baseline survival functions estimated in the training cohort were applied unchanged to the independent validation cohort. No predictor reselection, coefficient refitting, or recalibration was performed. Discrimination was assessed using Harrell’s C-index and time-dependent area under the curve (AUC) at 1 and 2 years. Time-dependent AUCs were estimated using inverse probability of censoring weighting. Calibration was evaluated at the same time points by comparing predicted PFS probabilities with Kaplan–Meier estimates of observed PFS.

Incremental discrimination was evaluated by applying the fixed Model A and Model B equations to the validation cohort. Between-model differences in C-index and time-dependent AUC were estimated using 5,000 paired bootstrap resamples with corresponding 95% CIs. Complete Model B coefficients, predictor coding, baseline survival estimates, and the equation for calculating individualized PFS probabilities are provided in Supplementary Table 5.

**Model Output, Class Imbalance, Fairness, and Model Updating**

Model B generates continuous individualized probabilities of remaining progression free at 1 and 2 years after ARPI initiation. No binary risk classification or clinical decision threshold was incorporated into the model. The data-derived 19.0-ng/mL 25(OH)D threshold was used only for descriptive Kaplan–Meier visualization and was not used to calculate model predictions or define treatment groups.

No class-imbalance correction, oversampling, undersampling, or synthetic sampling procedure was used because the primary prediction analysis was based on right-censored time-to-event Cox regression rather than binary classification.

The same eligibility criteria, predictor definitions, outcome definitions, and modeling equation were applied to the development and external validation cohorts. The cohorts differed geographically and institutionally, with Etlik City Hospital used for model development and Gazi University and Kütahya City Hospital used for external validation. No formal center-specific model updating or recalibration was performed. Given the limited number of participating centers and PFS events within individual external centers, heterogeneity in model performance across individual institutions was not formally estimated.

No formal algorithmic fairness adjustment was performed. Race or ethnicity, socioeconomic status, and other social determinants were not available in a sufficiently complete and harmonized form across centers to permit reliable subgroup-specific model-performance analyses. Consequently, fairness and transportability across these sociodemographic groups could not be evaluated and require assessment in future external validation studies.

**Treatment-Related Sensitivity and Interaction Analyses**

Treatment heterogeneity was examined by sequentially adding ARPI agent, treatment intensification strategy (doublet vs triplet therapy), and calendar year of ARPI initiation to Model B. A fully treatment-adjusted model incorporating all three variables was also fitted.

For patients receiving triplet therapy, docetaxel was planned at 75 mg/m² every 3 weeks for 6 cycles. The number of administered cycles, timing of the first docetaxel dose relative to ARPI initiation, completion of the planned 6-cycle course, dose reductions, treatment delays, and premature discontinuation were abstracted from institutional treatment records.

Potential effect modification by ARPI agent was explored by adding 25(OH)D-by-ARPI interaction terms to Model B, with enzalutamide as the reference category. Individual interaction HRs with 95% CIs and a global joint Wald test for interaction were reported. Because of the limited size of treatment-specific subgroups, these analyses were considered exploratory.

**Additional Sensitivity and Exploratory Analyses**

Season of baseline 25(OH)D measurement was determined from the recorded blood-collection date and categorized as winter (December–February), spring (March–May), summer (June–August), or autumn (September–November). Additional sensitivity analyses adjusted Model B for season of 25(OH)D measurement and baseline estimated glomerular filtration rate (eGFR), separately and simultaneously.

The proportional hazards assumption was evaluated using Schoenfeld residuals. Multicollinearity in the post-LASSO models was assessed using variance inflation factors.

The functional form of the association between baseline 25(OH)D and PFS was examined in the training cohort using restricted cubic spline analysis adjusted for the clinical predictors retained in Model A. Three knots were placed at 6.1, 19.0, and 31.8 ng/mL, with 19.0 ng/mL used as the reference value. Overall association and departure from linearity were evaluated separately.

Given the limited number of deaths, overall survival (OS) analyses were considered exploratory. The multivariable OS model was deliberately parsimonious and included continuous baseline 25(OH)D together with CHAARTED disease volume as a core clinical prognostic covariate. Additional OS covariates were not selected according to univariable p-values.

**Software and Computational Environment**

Statistical analyses were performed using IBM SPSS Statistics for Windows, version 25.0 (IBM Corp., Armonk, NY, USA), and Python, version 3.12 (Python Software Foundation). Data management and preprocessing were performed using pandas and NumPy. Statistical analyses were conducted using SciPy and statsmodels. Survival analyses were performed using lifelines, while penalized regression, model discrimination, and time-dependent performance analyses used scikit-learn and scikit-survival, as appropriate. Figures were generated using Matplotlib.

**Supplementary Table 1. Comparison of Baseline Characteristics According to Baseline Serum 25-Hydroxyvitamin D Level in the Overall Cohort**

| **Characteristic** | **25(OH)D ≤19.0 ng/mL (n=134)** | **25(OH)D>19.0 ng/mL (n=139)** | **p-value** |
| --- | --- | --- | --- |
| Baseline 25(OH)D, ng/mL, median (IQR) | 11.0 (8.0–14.0) | 27.0 (24.0–32.0) | — |
| Demographic and clinical characteristics |  |  |  |
| Age, years, median (IQR) | 71.0 (64.2–77.0) | 69.0 (64.0–75.0) | .061 |
| Age ≥65 years, n (%) | 100 (74.6) | 100 (71.9) | .616 |
| Body mass index, kg/m², median (IQR) | 26.4 (24.1–28.3) | 25.6 (23.9–27.9) | .151 |
| mCCI, median (IQR) | 9.0 (8.0–10.0) | 9.0 (8.0–11.0) | .963 |
| mCCI ≥8, n (%) | 119 (88.8) | 107 (77.0) | .010 |
| Smoking history present, n (%) | 95 (70.9) | 100 (71.9) | .848 |
| ECOG performance status ≥2, n (%) | 37 (27.6) | 22 (15.8) | .018 |
| ISUP grade group 4–5, n (%) | 104 (77.6) | 88 (63.3) | .010 |
| Disease characteristics |  |  |  |
| CHAARTED high-volume disease, n (%) | 83 (61.9) | 85 (61.2) | .893 |
| Bone metastasis present, n (%) | 115 (85.8) | 95 (68.3) | <.001 |
| Visceral metastasis present, n (%) | 34 (25.4) | 43 (30.9) | .307 |
| PSMA PET SUVmax, median (IQR) | 17.0 (8.9–23.0) | 10.0 (8.0–23.0) | .045 |
| Baseline laboratory characteristics |  |  |  |
| Baseline PSA, ng/mL, median (IQR) | 59.0 (23.0–329.0) | 23.0 (8.5–77.0) | <.001 |
| Hemoglobin, g/dL, median (IQR) | 13.4 (11.7–14.1) | 13.9 (13.5–15.0) | <.001 |
| White blood cell count, ×10³/µL, median (IQR) | 6.8 (5.8–8.1) | 7.6 (6.8–8.6) | <.001 |
| Absolute neutrophil count, ×10³/µL, median (IQR) | 4.0 (3.4–4.9) | 4.5 (3.9–5.0) | .056 |
| Absolute lymphocyte count, ×10³/µL, median (IQR) | 1.8 (1.3–2.1) | 2.1 (1.9–2.6) | <.001 |
| Platelet count, ×10³/µL, median (IQR) | 236.0 (196.5–281.5) | 240.0 (201.5–297.5) | .519 |
| NLR, median (IQR) | 2.5 (1.7–3.5) | 2.0 (1.8–2.3) | .008 |
| Serum albumin, g/L, median (IQR) | 42.0 (39.0–44.0) | 45.0 (44.0–46.0) | <.001 |
| C-reactive protein, mg/L, median (IQR) | 9.1 (5.8–16.2) | 10.7 (4.9–17.4) | .946 |
| Lactate dehydrogenase, U/L, median (IQR) | 193.0 (170.5–235.5) | 182.0 (169.0–202.0) | .017 |
| Alkaline phosphatase, U/L, median (IQR) | 114.5 (76.0–272.0) | 90.0 (78.5–111.0) | .003 |
| NCR score, median (IQR) | 113.9 (68.7–170.6) | 105.6 (60.6–223.9) | .549 |
| eGFR, mL/min/1.73 m², median (IQR) | 82 (68–94) | 84 (71–96) | .268 |
| Season of 25(OH)D measurement, n (%) |  |  | .003 |
| Winter | 49 (36.6) | 29 (20.9) |  |
| Spring | 40 (29.9) | 34 (24.5) |  |
| Summer | 20 (14.9) | 37 (26.6) |  |
| Autumn | 25 (18.7) | 39 (28.1) |  |
| Systemic treatment characteristics |  |  |  |
| ADT type |  |  | .042 |
| LHRH agonist | 105 (78.4) | 120 (86.3) |  |
| LHRH antagonist | 17 (12.7) | 16 (11.5) |  |
| Bilateral orchiectomy | 12 (9.0) | 3 (2.2) |  |
| ARPI agent |  |  | .499 |
| Enzalutamide | 61 (45.5) | 73 (52.5) |  |
| Abiraterone | 64 (47.8) | 57 (41.0) |  |
| Apalutamide | 9 (6.7) | 9 (6.5) |  |
| Treatment intensification |  |  | .342 |
| ADT + ARPI | 109 (81.3) | 119 (85.6) |  |
| Triplet therapy | 25 (18.7) | 20 (14.4) |  |

Abbreviations: 25(OH)D, 25-hydroxyvitamin D; ADT, androgen deprivation therapy; ARPI, androgen receptor pathway inhibitor; ECOG, Eastern Cooperative Oncology Group; eGFR, estimated glomerular filtration rate; IQR, interquartile range; ISUP, International Society of Urological Pathology; LHRH, luteinizing hormone-releasing hormone; mCCI, modified Charlson Comorbidity Index; NCR, Nutritional C-Reactive Protein Ratio; NLR, neutrophil-to-lymphocyte ratio; PET, positron emission tomography; PSA, prostate-specific antigen; PSMA, prostate-specific membrane antigen; SUVmax, maximum standardized uptake value.

Data are presented as median (IQR) for continuous variables and number (percentage) for categorical variables. Patients were categorized using the 19.0-ng/mL baseline 25(OH)D cut-off identified in the training cohort. Between-group comparisons were performed using the Mann–Whitney U test for continuous variables and the chi-square or Fisher exact test, as appropriate, for categorical variables. Post-treatment variables were not included because this table was intended to compare baseline characteristics potentially related to the observed prognostic association.

**Supplementary Table 2. Comparison of Observed Baseline Characteristics and Available Outcomes Between Patients Included in and Excluded From the Analytic Cohort**

| **Characteristic** | **Included cohort (n=273)** | **Excluded cohort (n=80)** | **Absolute SMD** | **p-value** |
| --- | --- | --- | --- | --- |
| Age, years, median (IQR) | 70 (64–77) | 71 (64–78) | 0.08 | 0.480 |
| ECOG performance status ≥2, n (%) | 59 (21.6) | 19 (23.8) | 0.05 | 0.680 |
| ISUP grade group 4–5, n (%) | 192 (70.3) | 55 (68.8) | 0.03 | 0.790 |
| High-volume disease, n (%) | 168 (61.5) | 52 (65.0) | 0.07 | 0.570 |
| Visceral metastasis, n (%) | 77 (28.2) | 25 (31.3) | 0.07 | 0.600 |
| Bone metastasis, n (%) | 210 (76.9) | 63 (78.8) | 0.05 | 0.730 |
| Baseline PSA, ng/mL, median (IQR) | 46 (10–148) | 51 (11–159) | 0.09* | 0.440 |
| Hemoglobin, g/dL, median (IQR) | 13.7 (12.4–14.6) | 13.5 (12.2–14.5) | 0.08 | 0.390 |
| Serum albumin, g/L, median (IQR) | 44 (41–45) | 43 (40–45) | 0.11 | 0.240 |
| Triplet therapy, n (%) | 45 (16.5) | 12 (15.0) | 0.04 | 0.750 |
| Abiraterone, n (%) | 121 (44.3) | 36 (45.0) | 0.01 | — |
| Enzalutamide, n (%) | 134 (49.1) | 38 (47.5) | 0.03 | 0.940† |
| Apalutamide, n (%) | 18 (6.6) | 6 (7.5) | 0.03 | — |
| 1-year PFS‡ | 88.1% | 85.9% | — | 0.460 |
| Death during available follow-up‡ | 37 (13.5) | 9/58 (15.5) | 0.06 | 0.680 |

Abbreviations: ARPI, androgen receptor pathway inhibitor; ECOG, Eastern Cooperative Oncology Group; IQR, interquartile range; ISUP, International Society of Urological Pathology; PFS, progression-free survival; PSA, prostate-specific antigen; SMD, standardized mean difference.

* SMD was calculated using log-transformed baseline PSA because of its right-skewed distribution.
† p-value represents the overall comparison across ARPI categories.
‡ Outcome comparisons among excluded patients were restricted to individuals with adequate outcome ascertainment; patients lacking sufficient follow-up were not assigned or imputed outcome events. Because excluded patients had incomplete records by definition, available-case denominators were used where necessary.
P-values were calculated using the Mann–Whitney U test for continuous variables and the Pearson chi-square or Fisher exact test for categorical variables. SMDs are presented as descriptive measures of covariate imbalance and were not used for significance testing.

**Supplementary Table 3. Candidate Predictors and Retention in the LASSO-Penalized Cox Model for Progression-Free Survival in the Training Cohort**

| **Candidate predictor** | **Coding/modeling** | **Retained at λmin** |
| --- | --- | --- |
| Age | Continuous, per year | Yes |
| ECOG performance status | ≥2 vs 0–1 | No |
| ISUP grade group | 4–5 vs 1–3 | Yes |
| CHAARTED disease volume | High vs low | Yes |
| Visceral metastasis | Present vs absent | No |
| Baseline log-PSA | Continuous, natural-log transformed | Yes |
| Hemoglobin | Continuous, g/dL | Yes |
| Alkaline phosphatase | Continuous, U/L | No |
| Lactate dehydrogenase | Continuous, U/L | No |
| Treatment intensification | Triplet therapy vs ADT + ARPI | No |

Abbreviations: ADT, androgen deprivation therapy; ALP, alkaline phosphatase; ARPI, androgen receptor pathway inhibitor; ECOG, Eastern Cooperative Oncology Group; ISUP, International Society of Urological Pathology; LASSO, least absolute shrinkage and selection operator; LDH, lactate dehydrogenase; PFS, progression-free survival; PSA, prostate-specific antigen; λmin, penalty parameter corresponding to the minimum cross-validated partial-likelihood deviance.

Ten candidate baseline clinical and laboratory predictors were specified before penalized model fitting. LASSO-penalized Cox regression was performed exclusively in the training cohort (n=192; 85 PFS events). Continuous predictors were standardized before penalized fitting. The penalty parameter (λ) was selected using 5-fold cross-validation based on partial-likelihood deviance, and λmin was defined as the value of λ that minimized the cross-validated partial-likelihood deviance. Predictors with nonzero coefficients at λmin were retained for Model A. Baseline 25(OH)D was not included in the penalized clinical predictor-selection procedure because its incremental prognostic contribution was evaluated subsequently by adding it to the retained clinical predictor set in Model B. Predictor retention was not based on univariable p-values.

**Supplementary Table 4. External Validation Performance of the Clinical Model and the Clinical-Plus-25(OH)D Model for Progression-Free Survival**

| **Performance measure** | **Model A** | **Model B** | **Difference, Model B − Model A** |
| --- | --- | --- | --- |
| Harrell C-index (95% CI) | 0.71 (0.64–0.78) | 0.76 (0.69–0.83) | +0.05 (0.01 to 0.10) |
| 1-year time-dependent AUC (95% CI) | 0.72 (0.61–0.83) | 0.78 (0.68–0.88) | +0.06 (0.01 to 0.12) |
| 2-year time-dependent AUC (95% CI) | 0.74 (0.64–0.84) | 0.80 (0.71–0.89) | +0.06 (0.01 to 0.12) |

Abbreviations: 25(OH)D, 25-hydroxyvitamin D; AUC, area under the receiver operating characteristic curve; CI, confidence interval; PFS, progression-free survival.

External validation was performed in the independent validation cohort (n=81; 27 PFS events) using the regression coefficients estimated in the training cohort without coefficient refitting, predictor reselection, or recalibration. Model A comprised age, ISUP grade group, CHAARTED disease volume, baseline log-transformed PSA, and hemoglobin. Model B comprised the identical clinical predictor set plus baseline 25(OH)D. Harrell C-index confidence intervals and between-model differences were estimated using 5,000 paired bootstrap resamples. Time-dependent AUCs at 1 and 2 years were estimated using inverse-probability-of-censoring weighting; corresponding 95% CIs and between-model differences were obtained using the same paired bootstrap procedure.

**Supplementary Table 5. Complete Model B Coefficients, Baseline Survival Estimates, and Calculation of Individualized Progression-Free Survival**

| **Predictor** | **Coding / unit** | **Regression coefficient (β)** | **HR (95% CI)** | **p-value** |
| --- | --- | --- | --- | --- |
| Age | Per 1-year increase | 0.04028 | 1.041 (1.015–1.068) | 0.002 |
| ISUP grade group | Grade groups 4–5 vs 1–3 | 0.18159 | 1.199 (0.651–2.209) | 0.560 |
| CHAARTED disease volume | High vs low | 0.47366 | 1.606 (0.970–2.658) | 0.065 |
| Baseline PSA | Natural-log transformed | 0.15956 | 1.173 (1.047–1.315) | 0.006 |
| Hemoglobin | Per 1-g/dL increase | −0.04001 | 0.961 (0.840–1.099) | 0.561 |
| Baseline 25(OH)D | Per 10-ng/mL increase | −0.45484 | 0.635 (0.488–0.825) | <0.001 |

**Baseline survival estimates**

| **Time point** | **S₀(t)** |
| --- | --- |
| 12 months | 0.993025 |
| 24 months | 0.976417 |

Abbreviations: 25(OH)D, 25-hydroxyvitamin D; CI, confidence interval; HR, hazard ratio; ISUP, International Society of Urological Pathology; PFS, progression-free survival; PSA, prostate-specific antigen.

Model B was fitted in the training cohort using the clinical predictors retained by LASSO at λmin together with continuous baseline 25(OH)D. ISUP grade group was coded as 0 for grade groups 1–3 and 1 for grade groups 4–5. CHAARTED disease volume was coded as 0 for low-volume and 1 for high-volume disease. Baseline PSA was entered as its natural logarithm, and baseline 25(OH)D was entered per 10-ng/mL increase. The reported baseline survival estimates correspond to the uncentered Cox model and are therefore used with the raw covariate values specified above; predictors should not be mean-centered when applying the following equation.

Linear predictor (LP):

LP = 0.04028 × Age + 0.18159 × ISUP + 0.47366 × CHAARTED + 0.15956 × ln(PSA) − 0.04001 × Hemoglobin − 0.45484 × [25(OH)D / 10]

Individual predicted PFS probability:

Predicted PFS(t) = S₀(t)^exp(LP)

Therefore:

12-month predicted PFS = 0.993025^exp(LP)

24-month predicted PFS = 0.976417^exp(LP)

The regression coefficients and baseline survival function were estimated in the training cohort and should be applied without coefficient refitting or recalibration when reproducing the reported Model B predictions.

**Supplementary Table 6. Seasonal and Renal-Function Characteristics and Sensitivity Analyses of the Association Between Baseline 25(OH)D and Progression-Free Survival**

**Panel A. Baseline season of 25(OH)D measurement and renal function according to baseline 25(OH)D group**

| **Characteristic** | **25(OH)D ≤19 ng/mL (n=134)** | **25(OH)D >19 ng/mL (n=139)** | **p-value** |
| --- | --- | --- | --- |
| **Season of 25(OH)D measurement, n (%)** |  |  | 0.003 |
| Winter (December–February) | 49 (36.6) | 29 (20.9) |  |
| Spring (March–May) | 40 (29.9) | 34 (24.5) |  |
| Summer (June–August) | 20 (14.9) | 37 (26.6) |  |
| Autumn (September–November) | 25 (18.7) | 39 (28.1) |  |
| Baseline eGFR, mL/min/1.73 m², median (IQR) | 82 (68–94) | 84 (71–96) | 0.268 |

**Panel B. Sensitivity analyses of the association between baseline 25(OH)D and progression-free survival**

| **Analysis** | **Model specification** | **n** | **HR per 10-ng/mL increase in 25(OH)D** | **95% CI** | **p-value** |
| --- | --- | --- | --- | --- | --- |
| Primary multivariable Cox model | Primary Model B | 273 | 0.63 | 0.49–0.83 | <0.001 |
| Stabilized inverse-probability-of-inclusion weighted model | Stabilized inclusion weighting | 273 | 0.65 | 0.50–0.85 | 0.002 |
| Prespecified clinical multivariable model | Prespecified clinical covariate adjustment | 273 | 0.66 | 0.51–0.86 | 0.002 |
| Season-adjusted model | Primary Model B + season of 25(OH)D measurement | 273 | 0.65 | 0.50–0.85 | 0.002 |
| Renal-function-adjusted model | Primary Model B + baseline eGFR | 273 | 0.64 | 0.49–0.84 | 0.001 |
| Season- and renal-function-adjusted model | Primary Model B + season of 25(OH)D measurement + baseline eGFR | 273 | 0.68 | 0.52–0.88 | 0.007 |

Abbreviations: CI, confidence interval; eGFR, estimated glomerular filtration rate; HR, hazard ratio; IQR, interquartile range; PFS, progression-free survival; 25(OH)D, 25-hydroxyvitamin D.

Season of blood collection was categorized as winter (December–February), spring (March–May), summer (June–August), and autumn (September–November). In Panel A, between-group comparisons were performed using the Pearson chi-square or Fisher exact test for categorical variables and the Mann–Whitney U test for continuous variables, as appropriate. The p-value for season represents the overall comparison across the four seasonal categories. In Panel B, HRs are reported per 10-ng/mL increase in baseline serum 25(OH)D. The stabilized inverse-probability-of-inclusion weighted analysis used weights estimated from baseline characteristics available among otherwise eligible patients. The prespecified clinical multivariable model is described in the Statistical Analysis section. Season- and renal-function-adjusted sensitivity models retained the covariates of the primary Model B and additionally included the variables specified above. HRs below 1.0 indicate a lower hazard of progression with higher baseline 25(OH)D.

**Supplementary Table 7. Triplet-Therapy Characteristics, Treatment-Adjusted Sensitivity Analyses, and Exploratory ARPI Interaction Analysis**

**Panel A. Treatment Characteristics Among Patients Receiving Triplet Therapy**

| **Characteristic** | **Triplet cohort (n=45)** |
| --- | --- |
| Docetaxel received | 45 (100%) |
| Planned docetaxel regimen | 75 mg/m² every 3 weeks for 6 cycles |
| Completed all 6 planned cycles | 41 (91.1%) |
| Completed ≥5 cycles | 43 (95.6%) |
| Number of docetaxel cycles, median (IQR) | 6 (6–6) |
| Docetaxel initiated within 30 days of ARPI initiation | 42 (93.3%) |
| Interval from ARPI initiation to first docetaxel, days, median (IQR) | 8 (3–14) |
| ≥1 docetaxel dose reduction | 6 (13.3%) |
| ≥1 treatment delay | 8 (17.8%) |
| Premature docetaxel discontinuation | 4 (8.9%) |

**Panel B. Sensitivity Analyses Accounting for Treatment Heterogeneity**

| **Model** | **25(OH)D HR per 10-ng/mL increase** | **95% CI** | **p-value** |
| --- | --- | --- | --- |
| Primary Model B | 0.64 | 0.49–0.83 | <0.001 |
| Model B + ARPI agent | 0.64 | 0.49–0.84 | 0.001 |
| Model B + treatment intensification | 0.63 | 0.48–0.83 | <0.001 |
| Model B + ARPI agent + treatment intensification | 0.64 | 0.49–0.84 | 0.001 |
| Model B + calendar year of ARPI initiation | 0.65 | 0.50–0.85 | 0.002 |
| Fully treatment-adjusted model* | 0.65 | 0.49–0.85 | 0.002 |

**Panel C. Exploratory Interaction Analysis Between Baseline 25(OH)D and ARPI Agent**

| **ARPI agent** | **n** | **PFS events** | **25(OH)D HR per 10-ng/mL increase (95% CI)** | **Interaction HR vs enzalutamide (95% CI)** | **Interaction p-value** |
| --- | --- | --- | --- | --- | --- |
| Enzalutamide | 90 | 32 | 0.59 (0.39–0.89) | Reference | — |
| Abiraterone | 86 | 48 | 0.72 (0.51–1.01) | 1.21 (0.72–2.03) | 0.477 |
| Apalutamide | 16 | 5 | 0.30 (0.10–0.92) | 0.51 (0.15–1.67) | 0.265 |
| Global interaction | — | — | — | — | 0.313 |

Abbreviations: 25(OH)D, 25-hydroxyvitamin D; ADT, androgen deprivation therapy; ARPI, androgen receptor pathway inhibitor; CI, confidence interval; HR, hazard ratio; IQR, interquartile range; ISUP, International Society of Urological Pathology; PFS, progression-free survival; PSA, prostate-specific antigen.

Data in Panel A are presented as n (%) unless otherwise specified. Triplet therapy was defined as ADT plus an ARPI plus docetaxel. Dose reductions and treatment delays were not mutually exclusive. Panel B presents sequential sensitivity analyses evaluating the robustness of the association between baseline 25(OH)D and PFS after accounting for treatment heterogeneity. Model B comprised the clinical predictors retained through penalized model development together with baseline 25(OH)D. Treatment-related covariates were added sequentially rather than selected according to statistical significance. The fully treatment-adjusted model additionally included ARPI agent, treatment intensification strategy (doublet vs triplet), and calendar year of ARPI initiation. Panel C presents an exploratory Cox proportional hazards interaction analysis conducted in the training cohort and including age, ISUP grade group, CHAARTED disease volume, baseline log-transformed PSA, hemoglobin, baseline 25(OH)D, ARPI agent, and 25(OH)D-by-ARPI interaction terms. Enzalutamide was the reference ARPI category, and 25(OH)D was modeled continuously per 10-ng/mL increase. The global interaction test jointly evaluated the 25(OH)D-by-abiraterone and 25(OH)D-by-apalutamide interaction terms. Agent-specific estimates should be interpreted cautiously, particularly for apalutamide because of the small subgroup size and limited number of PFS events.

**Supplementary Table 8. Exploratory Univariable and Parsimonious Multivariable Cox Regression Analyses for Overall Survival in the Training Cohort**

| Variables | Univariate Analysis |  | Multivariate Analysis* |  |
| --- | --- | --- | --- | --- |
|  | **HR (95% CI)** | **p-value** | **HR (95% CI)** | **p-value** |
| Age (per 1-year increase) | 1.013 (0.972–1.057) | 0.536 | - | - |
| mCCI Score Category (≥8 vs. <8) | 1.462 (0.432–4.944) | 0.541 | - | - |
| Smoking History (Present vs. Absent) | 1.052 (0.444–2.493) | 0.908 | - | - |
| ECOG Performance Status (≥2 vs. 0-1) | 1.147 (0.484–2.719) | 0.755 | - | - |
| ISUP Grade Group | 1.248 (0.907–1.716) | 0.174 | - | - |
| Disease volume (High vs. Low) | 1.995 (0.882–4.512) | 0.097 | 1.711 (0.755–3.876) | 0.198 |
| Visceral Metastasis (Present vs. Absent) | 1.099 (0.457–2.643) | 0.832 | - | - |
| Baseline log (PSA) (per 1-unit increase in log-PSA) | 1.109 (0.922–1.333) | 0.271 | - | - |
| Baseline 25(OH)D, per 10-ng/mL increase | **0.624 (0.407–0.951)** | **0.029** | **0.651 (0.420–0.991)** | **0.048** |
| log(NCR) Score (per 1-unit increase in log-NCR) | 0.756 (0.472–1.209) | 0.243 | - | - |
| Hemoglobin (g/dL) | 0.844 (0.682–1.043) | 0.116 | - | - |
| Neutrophil-to-Lymphocyte Ratio (NLR) | 1.010 (0.958–1.064) | 0.723 | - | - |
| Lactate Dehydrogenase (U/L) | 1.001 (0.999–1.004) | 0.342 | - | - |
| Alkaline Phosphatase (U/L) | 1.000 (1.000–1.001) | 0.345 | - | - |
| Treatment Intensification (Triplet) | 1.224 (0.514–2.912) | 0.648 | - | - |
| ARPI Agent Type | 1.637 (0.832–3.218) | 0.153 | - | - |

Abbreviations: ARPI, Androgen Receptor Pathway Inhibitor; CI, Confidence Interval; ECOG, Eastern Cooperative Oncology Group; HR, Hazard Ratio; ISUP, International Society of Urological Pathology; mCCI, modified Charlson Comorbidity Index; NCR, Nutritional C-Reactive Protein Ratio; PSA, Prostate-Specific Antigen.

* Because only 27 deaths occurred in the training cohort, all OS analyses were considered exploratory. To limit model complexity and reduce the risk of overfitting, a deliberately parsimonious multivariable model was used that included baseline 25(OH)D together with CHAARTED disease volume as a core clinical prognostic covariate. No additional covariates were selected on the basis of univariable p-values. The exploratory OS model demonstrated a C-index of 0.659 in the training cohort and 0.775 in the validation cohort.

**Supplementary Table 9. Post-treatment PSA Response Characteristics in the Training and External Validation Cohorts**

| **Characteristic** | **Total Cohort (n=273)** | **Training Cohort (n=192)** | **External Validation Cohort (n=81)** | **p-value** |
| --- | --- | --- | --- | --- |
| Nadir PSA, ng/mL, median (IQR) | 0.1 (0.0–0.9) | 0.1 (0.0–1.5) | 0.1 (0.0–0.3) | 0.022 |
| 1-month PSA, ng/mL, median (IQR) | 3.4 (1.0–34.0) | 3.3 (1.0–40.0) | 4.0 (0.8–19.0) | 0.318 |
| PSA decline ≥50%, n (%) | 235 (86.1) | 162 (84.4) | 73 (90.1) | 0.253 |
| PSA decline ≥90%, n (%) | 208 (76.2) | 141 (73.4) | 67 (82.7) | 0.120 |

Abbreviations: IQR, interquartile range; PSA, prostate-specific antigen.

Post-treatment PSA variables are presented descriptively and were not considered candidate predictors for baseline prognostic model development because they were measured after treatment initiation. P-values compare the training and external validation cohorts and were calculated using the Mann–Whitney U test for continuous variables and the Pearson chi-square or Fisher exact test for categorical variables, as appropriate. Statistically significant p-values (<0.05) are shown in bold.

**Supplementary Figure 1. Kaplan–Meier survival curves for progression-free survival (A) and overall survival (B) according to baseline serum 25-hydroxyvitamin D levels in the external validation cohort**

**Abbreviations:** CI, confidence interval; NR, not reached; OS, overall survival; PFS, progression-free survival.

The 19.0-ng/mL cut-off identified using maximally selected rank statistics in the training cohort was applied unchanged to the external validation cohort. Patients in the low 25(OH)D group (≤19.0 ng/mL) had a median PFS of 34.7 months (95% CI, 27.2–39.6), whereas median PFS was not reached (NR) in the high 25(OH)D group (>19.0 ng/mL; 95% CI, 25.0–NR) (log-rank p=0.018). Median OS was 49.9 months (95% CI, 41.3–52.5) in the low 25(OH)D group and was not reached in the high 25(OH)D group (95% CI, 28.7–NR) (log-rank p=0.042). Numbers at risk are shown below each panel, and tick marks on the survival curves indicate censored observations.

**Alt text:** Two-panel Kaplan–Meier figure showing progression-free survival and overall survival in the external validation cohort, stratified by the 19.0-ng/mL baseline 25(OH)D cut-off derived from the training cohort. Survival curves compare patients with 25(OH)D ≤19.0 ng/mL and>19.0 ng/mL, with censoring marks and numbers at risk displayed over follow-up.

**Supplementary Figure 2. Time-Dependent Receiver Operating Characteristic Curves Comparing the Clinical Model and the Clinical-Plus-25(OH)D Model for Progression-Free Survival**

Abbreviations: 25(OH)D, 25-hydroxyvitamin D; AUC, area under the receiver operating characteristic curve; CI, confidence interval; PFS, progression-free survival; ROC, receiver operating characteristic.

Time-dependent ROC analyses compared Model A and Model B for prediction of PFS. Panel A shows 1-year PFS discrimination in the training cohort, and Panel B shows 2-year PFS discrimination in the training cohort. Panels C and D show the corresponding 1-year and 2-year analyses in the external validation cohort, respectively. Model A comprised age, ISUP grade group, CHAARTED disease volume, baseline log-transformed PSA, and hemoglobin. Model B comprised the identical clinical predictor set plus baseline 25(OH)D. Time-dependent AUCs were estimated using inverse-probability-of-censoring weighting. In the external validation cohort, the regression coefficients estimated in the training cohort were applied without coefficient refitting, predictor reselection, or recalibration. Confidence intervals were estimated using paired bootstrap resampling.

**Alt text:** Four-panel time-dependent ROC figure comparing Model A and Model B for progression-free survival prediction. Panels A and B show 1- and 2-year discrimination in the training cohort, and Panels C and D show the corresponding analyses in the external validation cohort. Model B, which additionally includes baseline 25(OH)D, is compared with the clinical-only Model A using time-dependent AUC estimates.

**Supplementary Figure 3. Calibration of the Clinical Model and the Clinical-Plus-25(OH)D Model for 1- and 2-Year Progression-Free Survival in the Training Cohort**

Abbreviations: 25(OH)D, 25-hydroxyvitamin D; PFS, progression-free survival.

Calibration plots compare predicted and observed PFS probabilities in the training cohort. Panel A shows 1-year calibration for Model A, which comprised the clinical predictors retained by LASSO-penalized Cox regression. Panel B shows 1-year calibration for Model B, which comprised the identical clinical predictor set plus baseline 25(OH)D. Panels C and D show the corresponding 2-year calibration plots for Model A and Model B, respectively. Observed PFS probabilities were estimated using the Kaplan–Meier method within groups defined by predicted risk. The dashed diagonal line represents perfect agreement between predicted and observed probabilities.

**Alt text:** Four-panel calibration figure comparing predicted and observed progression-free survival probabilities for Model A and Model B in the training cohort. Panels A and B show 1-year calibration, and Panels C and D show 2-year calibration. The dashed diagonal line represents perfect agreement between predicted and observed probabilities.

**Supplementary Figure 4. External Validation Calibration of the Clinical Model and the Clinical-Plus-25(OH)D Model for 1- and 2-Year Progression-Free Survival**

**Abbreviations:** 25(OH)D, 25-hydroxyvitamin D; CI, confidence interval; PFS, progression-free survival.

Calibration was assessed in the external validation cohort (n=81; 27 PFS events) using the regression coefficients and baseline survival functions estimated in the training cohort, without coefficient refitting, predictor reselection, or recalibration. Panel A shows 1-year calibration for Model A, and Panel B shows 1-year calibration for Model B. Panels C and D show the corresponding 2-year calibration plots for Model A and Model B, respectively. Model A comprised age, ISUP grade group, CHAARTED disease volume, baseline log-transformed PSA, and hemoglobin. Model B comprised the identical clinical predictor set plus baseline 25(OH)D. Predicted PFS probabilities were grouped into quintiles, and observed PFS probabilities were estimated using the Kaplan–Meier method. Vertical error bars represent 95% confidence intervals for the observed Kaplan–Meier estimates calculated using Greenwood variance with log-log transformation. The dashed diagonal line represents perfect agreement between predicted and observed probabilities.

**Alt text:** Four-panel calibration figure showing agreement between predicted and observed progression-free survival probabilities in the external validation cohort. Panels A and B show 1-year calibration for Model A and Model B, respectively, and Panels C and D show the corresponding 2-year calibration. Points represent grouped predicted-risk estimates with 95% confidence intervals for observed Kaplan–Meier probabilities, and the dashed diagonal line represents perfect calibration.

**Supplementary Figure 5. Decision Curve Analysis Comparing the Clinical Model and the Clinical-Plus-25(OH)D Model for 1- and 2-Year Progression-Free Survival in the Training Cohort**

Abbreviations: 25(OH)D, 25-hydroxyvitamin D; DCA, decision curve analysis; PFS, progression-free survival.

Decision curve analysis was performed in the training cohort to compare the net benefit of Model A and Model B across threshold probabilities from 0.05 to 0.50. Panel A shows the analysis for 1-year PFS, and Panel B shows the corresponding analysis for 2-year PFS. Model A comprised the clinical predictors retained by LASSO-penalized Cox regression, whereas Model B comprised the identical clinical predictor set plus baseline 25(OH)D. The “Treat all” and “Treat none” strategies are shown as reference approaches. Net benefit was estimated using time-dependent inverse-probability-of-censoring weighting to account for censoring before the specified time horizon.

**Alt text:** Two-panel decision curve analysis comparing the net benefit of Model A and Model B for prediction of 1-year and 2-year progression-free survival in the training cohort. Panel A shows 1-year PFS and Panel B shows 2-year PFS across threshold probabilities from 0.05 to 0.50, with “Treat all” and “Treat none” shown as reference strategies.

**Supplementary Figure 6. Restricted Cubic Spline Analysis of the Association Between Baseline 25(OH)D and Progression-Free Survival**

Abbreviations: 25(OH)D, 25-hydroxyvitamin D; CI, confidence interval; HR, hazard ratio; PFS, progression-free survival; RCS, restricted cubic spline.

Restricted cubic spline analysis was performed in the training cohort (n=192; 85 PFS events) to evaluate the shape of the association between baseline 25(OH)D and progression risk. The spline model was adjusted for the clinical predictors retained in Model A: age, ISUP grade group, CHAARTED disease volume, baseline log-transformed PSA, and hemoglobin. Three knots were placed at the 10th, 50th, and 90th percentiles of baseline 25(OH)D (6.1, 19.0, and 31.8 ng/mL, respectively), with the median value of 19.0 ng/mL used as the reference. The solid line represents the estimated HR, the shaded area represents the 95% CI, the horizontal dashed line indicates an HR of 1.0, and the vertical dotted line indicates the reference value. The overall association was statistically significant (*P* overall = 0.001), with no evidence of significant nonlinearity (*P* for nonlinearity = 0.327).

**Alt text:** Restricted cubic spline plot showing the adjusted association between baseline 25(OH)D and progression risk in the training cohort. The solid curve represents the estimated hazard ratio across 25(OH)D values, the shaded band represents the 95% confidence interval, the horizontal dashed line marks a hazard ratio of 1.0, and the vertical dotted line marks the 19.0-ng/mL reference value.
